# Computational Prediction of an Antimony-Based n-Type Transparent Conducting Oxide: F-Doped Sb_2_O_5_

**DOI:** 10.1021/acs.chemmater.3c03257

**Published:** 2024-03-11

**Authors:** Ke Li, Joe Willis, Seán R. Kavanagh, David O. Scanlon

**Affiliations:** † Department of Chemistry, University College London, 20 Gordon Street, London WC1H 0AJ, U.K.; ‡ Thomas Young Centre, University College London, Gower Street, London WC1E 6BT, U.K.; § Department of Materials, Imperial College London, Exhibition Road, London SW7 2AZ, U.K.; ∥ School of Chemistry, University of Birmingham, Edgbaston, Birmingham B15 2TT, U.K.

## Abstract

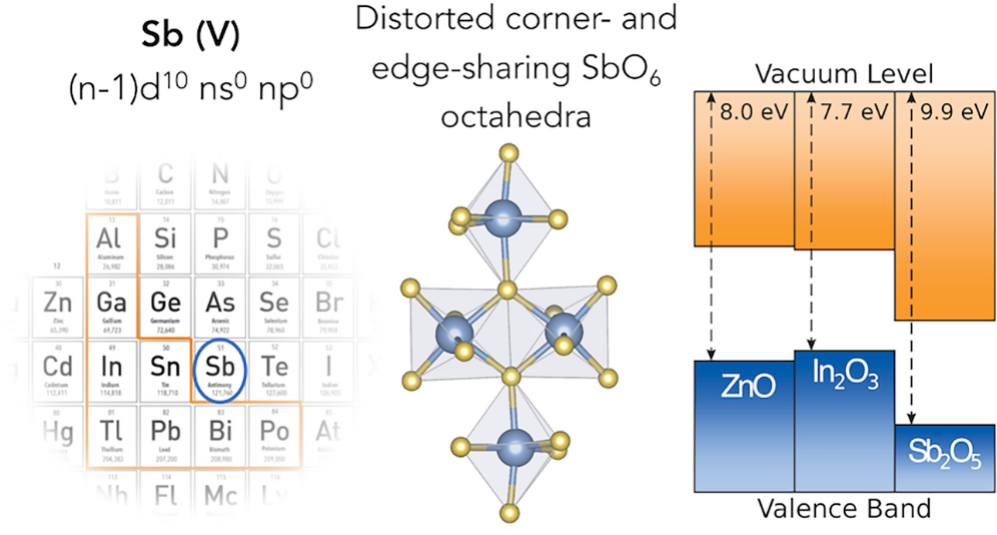

Transparent conducting oxides (TCOs) possess a unique combination of optical transparency and electrical conductivity, making them indispensable in optoelectronic applications. However, their heavy dependence on a small number of established materials limits the range of devices that they can support. The discovery and development of additional wide bandgap oxides that can be doped to exhibit metallic-like conductivity are therefore necessary. In this work, we use hybrid density functional theory to identify a binary Sb(V) system, Sb_2_O_5_, as a promising TCO with high conductivity and transparency when doped with fluorine. We conducted a full point defect analysis, finding F-doped Sb_2_O_5_ to exhibit degenerate n-type transparent conducting behavior. The inherently large electron affinity found in antimony oxides also widens their application in organic solar cells. Following our previous work on zinc antimonate, this work provides additional support for designing Sb(V)-based oxides as cost-effective TCOs for a broader range of applications.

## Introduction

In the field of modern (opto-)electronics, transparent conducting oxides (TCOs) act as pivotal materials, bridging the gap between conductivity and optical transparency.^1,2^ From touchscreen devices that can interact with users to solar cells that harvest sunlight energy, the pursuit of high-performance TCOs continues to contribute to a variety of technological applications, yielding more efficient devices and reduced energy consumption.

The developmental history of TCOs spans several decades. Cadmium oxide (CdO), the first recorded TCO, was introduced by Bädeker in 1907.^3^ It was found that a low resistivity of 1.20 × 10^–3^ Ωcm and a large mobility of over 100 cm^2^ V^–1^ s^–1^ at a high carrier concentration of 1 × 10^21^ cm^–3^ can be achieved in CdO.^4^ Meanwhile, the Moss–Burstein shift leads to a wide optical bandgap, enabling its high level of transparency.^5^ Despite the toxic nature of CdO, this discovery laid the foundation and opened up a research area for improved TCOs. Since then, a number of n-type TCOs have been established, including In_2_O_3_, Ga_2_O_3_, SnO_2_, and ZnO.^6−9^ Among these metal oxides, In_2_O_3_ is the most popular transparent conductor, widely used in cutting-edge technology due to its high conductivity and over 90% transparency.^1^ However, the high cost due to the scarcity of indium raises concerns about its long-term sustainability in large-scale applications. The development of new promising TCOs is therefore driven by the demand for cost efficiency, a broader application scope, and enhanced performance.^2^

A recurring characteristic observed among these successful n-type TCOs is the presence of post-transition metal cations with an electronic configuration of (*n* – 1)d^10^*n*s^0^*n*p^0^. A wide bandgap with highly dispersed conduction bands often exists in these oxides due to the strong antibonding hybridization of the valence cation s and the O 2p orbitals. This electronic configuration is primarily found in elements from groups 12, 13, and 14 in the periodic table. However, ZnSb_2_O_6_ has been recently proposed as an Sb(V)-based oxide with promising transparent conducting behavior, unusually deep band edges, and an interesting band alignment for optoelectronic applications.^10−14^ Sb is a group 15 element, but in its highest oxidation state [Sb(V)], it adopts the same (*n* – 1)d^10^*n*s^0^*n*p^0^ electron configuration as conventional post-transition metal TCOs. Furthermore, the increased abundance of Sb can contribute to a reduction in manufacturing costs.^15^ The discovery of this material not only brings valuable diversity to the field but also opens the possibility of Sb(V)-based oxides as lower-cost alternative TCOs. Following the success of ZnSb_2_O_6_, we consider the underexplored binary system Sb_2_O_5_ as a potential candidate due to its wide bandgap and comparable electronic structure to established metal oxide TCOs. The crystal structure of monoclinic Sb_2_O_5_ was first studied by Jansen in 1978, where Sb_2_O_5_ powder was synthesized by heating Sb_2_O_3_ in a steel autoclave that was charged with liquid oxygen.^16^ The electronic structure of Sb_2_O_5_ was briefly investigated along with its competing phases by Allen et al., while its properties as a potential TCO have been overlooked.^17^

In this work, we initially compared the formation energies of all reported polymorphs of Sb_2_O_5_ (Table S1), where monoclinic *C*2/*c* Sb_2_O_5_ was found to be the most stable crystal structure. We examined the crystal and electronic structures of monoclinic Sb_2_O_5_ with hybrid density functional theory (DFT). We find that Sb_2_O_5_ possesses a wide optical bandgap over 3.6 eV, suggesting visible light transparency, and high dispersion in the conduction band, suggesting good electron mobility. Charge transport properties were also evaluated, in which intrinsic Sb_2_O_5_ was found to exhibit relatively high electron mobility and thus conductivity—if large carrier concentrations can be achieved through extrinsic doping. To investigate the dopability of Sb_2_O_5_, we conducted a full intrinsic point defect analysis, finding the self-consistent Fermi level to reside deep within the bandgap and thus insulating behavior for undoped Sb_2_O_5_. To enhance its electrical conductivity, we considered introducing an extrinsic dopant to the oxygen site, namely, fluorine (F). Our investigation demonstrated that the fluorine-on-oxygen substitution F_O_ serves as a good electron donor in Sb_2_O_5_, shifting the self-consistent Fermi level into the conduction band and achieving equilibrium charge carrier concentrations on the order of 10^19^ cm^–3^—surpassing the Mott criterion for degenerate conductivity. Thus, we predict F-doped Sb_2_O_5_ to be a degenerate n-type TCO. The earth-abundant nature of antimony also renders F-doped Sb_2_O_5_ a promising candidate for achieving cost efficiency in optoelectronics applications. The large electron affinity (EA) observed in the band alignment of Sb_2_O_5_ extends the range of potential applications for TCOs. This study further demonstrates the feasibility and promising potential of designing Sb(V)-oxides as TCOs for lower cost and broader applications.

## Computational Methods

All DFT calculations were performed using the Vienna ab initio simulation package (VASP) code.^18−23^ Density functional perturbation theory and phonon dispersion calculations were performed using the Perdew–Burke–Ernzerhof for solids (PBEsol) exchange–correlation functional, which adopts the generalized gradient approximation.^24^ The rest of the calculations were performed using the PBE0 hybrid functional, which has been shown to accurately predict the electronic structure of Sb(V)-based oxides.^10,25^ Plane-wave cutoff and *k*-point sampling were tested and converged using vaspup2.0,^26^ with a cutoff energy of 500 eV and a *k*-point sampling of 6 × 6 × 5 for the 14-atom primitive cell of Sb_2_O_5_ found to be sufficient for total energies converged to 1 meV/atom. The crystal structure of Sb_2_O_5_ was visualized using vesta.^27^ The electronic band structure and density of states (DOS) were plotted using the sumo package.^28^ Charge transport properties were obtained and plotted using AMSET and ThermoParser.^29,30^ Phonon dispersion curves were calculated using PHONOPY and plotted using ThermoParser.^30,31^ The band alignment of bulk Sb_2_O_5_ was calculated using the core and vacuum energies from a 30 Å thick (001) surface slab with 20 Å of vacuum using the SURFAXE package.^32^

For defect calculations, an 112-atom supercell from a 1 × 2 × 2 expansion of the Sb_2_O_5_ conventional cell was used to minimize the interactions between periodic defects. To sample the defect configuration landscape and search for ground and metastable defect structures, the ShakeNBreak approach was used to initially generate Γ-point-only relaxations for each defect with 10 different local distortions.^33,34^ The ground-state structure found in these initial relaxations was then selected for structural optimization with a converged Γ-centered 2 × 2 × 2 *k*-point mesh to obtain the total energy of the ground-state defective supercell. The formation energy of a defect with the charge state *q* can be calculated using the equation below^35^
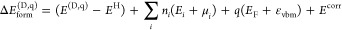
1where the first part indicates the energy difference between the defective supercell with the charge state q [*E*^(D,q)^] and the host supercell (*E*^H^), the second term represents the change in Gibbs free energy when adding or removing an atom from the supercell (*n*_*i*_ is the number of atoms, *E*_*i*_ is the elemental reference energy for the corresponding atom, and μ_*i*_ is the formal chemical potential), and the third term is a combination of self-consistent Fermi levels (*E*_F_) referenced to the valence band maximum (VBM) and the eigenvalue of the host VBM (ε_vbm_).^35−39^ An additional correction term (*E*^corr^), specifically the image-charge correction, is also needed to account for spurious finite-size supercell effects. Each term in eq 1 along with the formation energies of all intrinsic point defects and extrinsic defects are summarized in Tables S8 and S9. The Doped Python package was used to manage all the defect calculations and analysis.^40^ Data produced during this work are freely available at doi.org/10.5281/zenodo.10075593.

## Results and Discussion

### Crystal Structure

**Figure 1:**
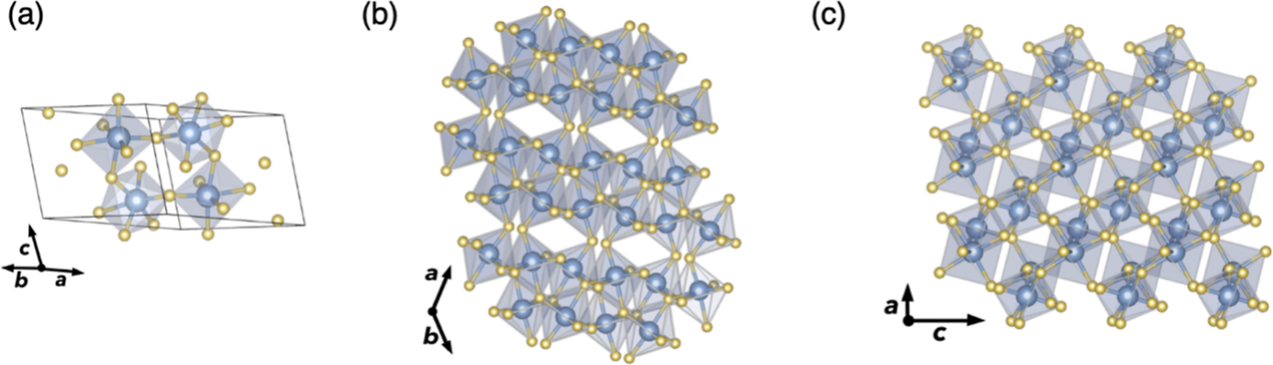
(a) Primitive unit cell of Sb_2_O_5_, where Sb atoms are in blue and O atoms in yellow. (b,c) show the Sb_2_O_5_ crystal structure looking down the *c* and *b* axes, respectively, showing the corner- and edge-sharing SbO_6_ octahedra.

Sb_2_O_5_ crystallizes in a monoclinic structure with the *C*2/*c* space group (Figure 1). Each Sb atom is surrounded by six oxygen atoms, forming a mixture of distorted SbO_6_ corner- and edge-sharing octahedra, yielding an infinite three-dimensional framework. Three crystallographically distinct oxygen sites exist, with one edge-sharing and two corner-sharing environments. The edge-sharing oxygen has *C*_1_ point symmetry and forms three different O–Sb bonds with an average bond length of 2.07 Å. Between the corner-sharing sites, one exhibits two identical O–Sb bonds with a length of 1.91 Å, yielding *C*_2_ symmetry, while the other has two nonequivalent O–Sb bond lengths of 1.92 and 1.89 Å, exhibiting *C*_1_ symmetry. As shown in Table 1, the calculated PBE0 lattice parameters are in excellent agreement with the experimental values.^16^ PBEsol- and HSE06-relaxed crystal structures were also compared (Table S2), where the PBEsol functional slightly overestimates the lattice parameters by around 2%, while the HSE06 lattice parameters are basically the same as those of PBE0. The phonon dispersion was also calculated (as shown in Figure S1 in the Supporting Information) and no imaginary modes were witnessed, demonstrating the dynamic stability of this compound.

**Table 1:** Comparison between the Conventional Crystal Lattice Parameters Calculated Using the PBE0 DFT Functional and the Experimental Values Measured Using Powder X-ray Diffraction.^16^

parameters	PBE0	experiment^16^	percentage difference (%)
*a*/Å	12.63	12.65	–0.10
*b*/Å	4.78	4.78	–0.02
*c*/Å	5.43	5.42	0.02
β/°	103.78	103.93	–0.14
volume/Å^3^	318.23	318.35	–0.04

### Electronic Structure

**Figure 2:**
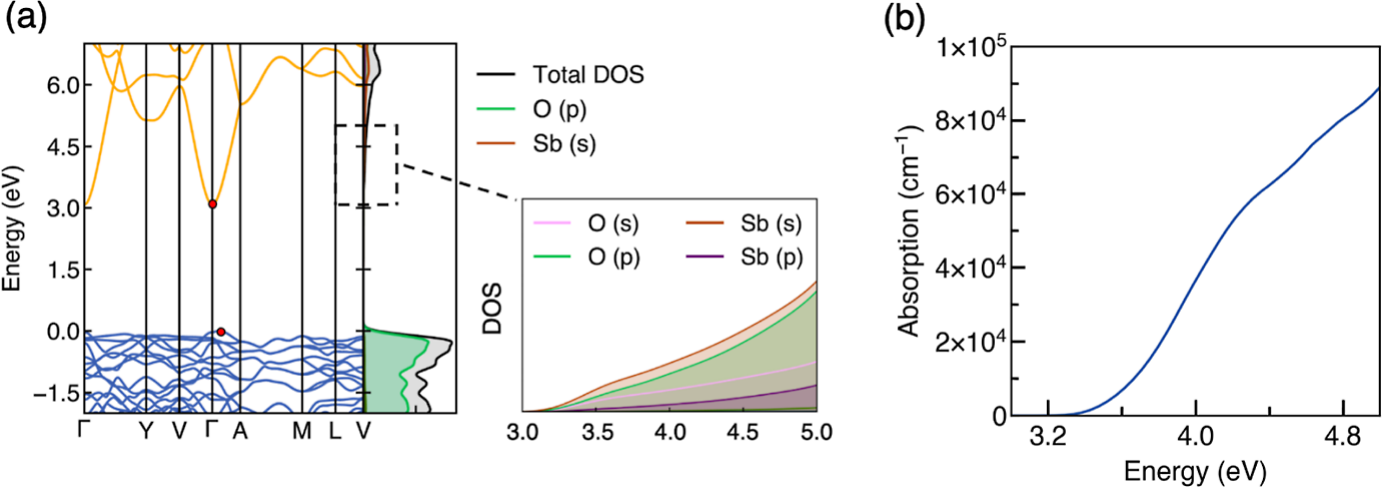
(a) Electronic band structure and orbital-decomposed DOS of Sb_2_O_5_ calculated using hybrid DFT (PBE0), where the conduction bands are shown in yellow and the valence bands in blue. (b) Band–band optical absorption for Sb_2_O_5_ plotted using SUMO.^28^

The PBE0-calculated electronic band structure of Sb_2_O_5_ in Figure 2a shows a slightly indirect bandgap of 3.08 eV, from the VBM located between Γ and A [0, 0, 0.5] to the conduction band minimum (CBM) at Γ. It has a direct bandgap of 3.11 eV at Γ. The conduction band is highly dispersed and has electron effective masses of 0.30, 0.30, and 0.34 *m*_e_ along the Γ → Y, Γ → V, and Γ → A directions, respectively. This results in a mean effective mass of 0.31 *m*_e_ for Sb_2_O_5_, indicating the potential for high electron mobility. The high dispersion of the conduction band arises due to the strong antibonding interaction of the unoccupied cation s orbitals and O 2p states as in other post-transition metal oxides, which can be seen from the crystal orbital Hamilton population (COHP) analysis shown in Figure S3. A flat valence band is observed, reflecting the localized character of the O 2p bonding states which dominate the VBM. This results in large hole effective masses of 1.48 and 1.84 *m*_e_ from VBM to A and VBM to Γ, respectively. The optical properties are shown in the optical absorption and Tauc plots (Figures 2b and S2), where the direct band-to-band absorption spectrum was calculated by using the PBE0 hybrid DFT functional. The absorption coefficient only reaches 10^4^ cm^–1^ at an energy of 3.6 eV. Indeed, an effective optical bandgap of ∼3.6 eV is predicted by determining the point at which the linear fit intersects the *x*-axis in the direct-gap Tauc plot (Figure S2). We note that this aligns closely with the band gap of ∼3.55 eV from optical transmittance measurements by Mindil et al. on cubic Sb_2_O_5_ nanorod films;^41^ however, no experimental measurement for *C*2/*c* monoclinic Sb_2_O_5_ is known. Sb_2_O_5_ has a centrosymmetric (*C*2/*c*) crystal structure, and so, it is likely that some of the low-energy optical transitions are symmetry-forbidden as in In_2_O_3_,^42^ resulting in weak absorption just above the direct bandgap.^43^

### Charge Transport Properties

For an ideal TCO, high carrier mobility is required to achieve optimum conductivity. The Mott criterion (*n*_Mott_) describes the critical carrier concentration at which a material is expected to exhibit metallic-like conductivity, given by^10,44−46^
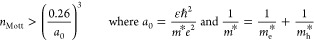
2where *a*_0_ is the effective Bohr radius (18.9 Å for Sb_2_O_5_); ε is the total dielectric constant, which is equal to the calculated total relative dielectric constant ([13.99, 12.16, 11.61]) times the permittivity of free space (ε_0_); and m* is the reduced effective mass. Using the electron and hole effective masses obtained from the Sb_2_O_5_ band structure, a reduced effective mass of 0.263 *m*_0_ was determined, showing a comparable value to that of traditional TCOs (0.13–0.55 *m*_0_).^47,48^ The Mott criterion for Sb_2_O_5_ predicts a critical carrier concentration of 1.09 × 10^18^ cm^–3^ based on these values, closely aligning with the conventional TCOs such as In_2_O_3_, SnO_2_, and Ga_2_O_3_, which have Mott criterion concentrations ranging from 1 × 10^18^ cm^–3^ to 3 × 10^18^ cm^–3^.^48−50^

**Figure 3:**
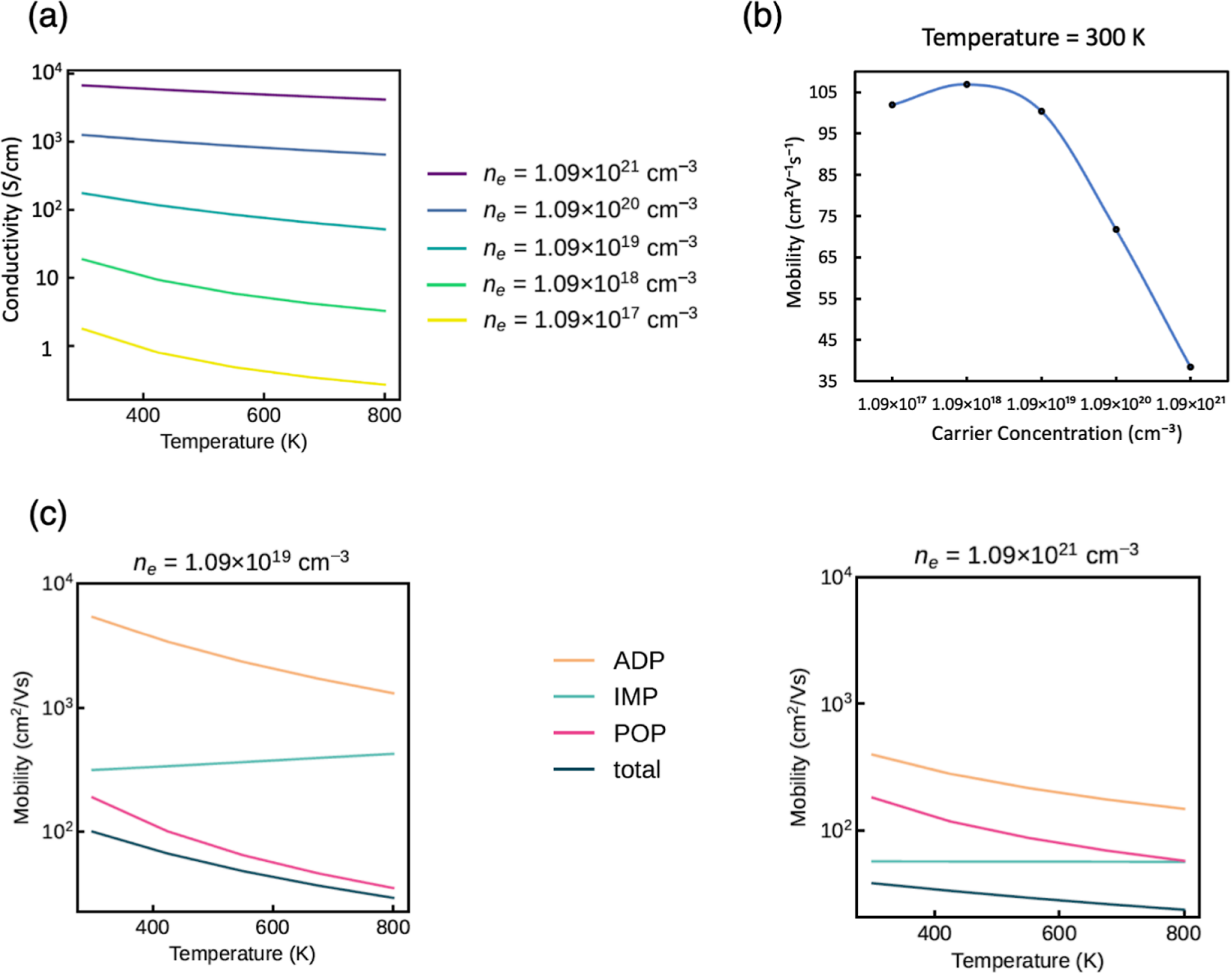
(a) Simulated conductivity of Sb_2_O_5_ over a range of carrier concentrations chosen with respect to the Mott criterion. (b) Calculated electron mobility of Sb_2_O_5_ over a range of carrier concentrations at room temperature. (c) Effect of scattering mechanisms on mobility at low and high carrier concentrations. ADP is acoustic deformation potential scattering, IMP is ionized impurity scattering, and POP is polar-optical phonon scattering.

The charge transport properties are calculated using AMSET, with the results shown in Figure 3.^29^Figure 3a illustrates how the conductivity changes with temperature at different carrier concentrations, from 1 order of magnitude smaller to 3 orders larger than the Mott criterion value. The conductivity is more sensitive to temperature at lower carrier concentrations, with conductivity decreasing as temperature increases—as expected for band-like carrier transport. At carrier concentrations of 10^19^ cm^–3^, the conductivity can reach over 100 S cm^–1^ at room temperature, which can be increased to just under 10^4^ S cm^–1^ at carrier concentrations of 10^21^ cm^–3^. Therefore, to achieve sufficient conductivity for TCO applications, high doping concentrations are needed in this system. The change in mobility with carrier concentrations at room temperature is shown in Figure 3b. A maximum mobility of around 106 cm^2^ V^–1^ s^–1^ is obtained at low carrier concentrations, while it declines with higher carrier concentrations. The typical mobility for n-type TCOs is from 50 to 70 cm^2^ V^–1^ s^–1^, and so, Sb_2_O_5_ has a competitive mobility compared with that of established TCOs.^51^

The analysis of the limiting scattering type for mobility is shown in Figure 3c. Polar-optical phonon (POP) is the dominant limiting factor for carrier concentrations below 10^20^ cm^–3^ across all temperatures above 300 K. For carrier concentrations over 10^20^ cm^–3^, the limiting scattering type becomes an ionized impurity (IMP) at temperatures lower than 800 K. In both regimes, the overall mobility decreases as temperature increases due to increased carrier-phonon (POP) scattering.

### Intrinsic Defect Chemistry

Defect chemistry is critical for tailoring the electrical conductivity and optical properties of the TCOs. As shown above, to achieve sufficiently high conductivities for TCO applications, high doping concentrations are required for this material. Thus, to investigate the dopability of this compound, a thorough study of the intrinsic defect chemistry was performed. Chemical potential limits need to be analyzed first as they provide a thermodynamic reference for the formation energy and stability of defects in materials. Table S5 lists the calculated chemical potential limits using the total energy per atom for each competing phase shown in Tables S3 and S4. Figure S4 shows the thermostability region of Sb_2_O_5_ where it exhibits a relatively wide Sb chemical potential range of around 2 eV and a smaller oxygen chemical potential range of 0.76 eV.

The total dielectric constant, used to obtain the finite-size correction term *E*^corr^ in eq 1, was calculated to be 13.99, 12.16, and 11.61 ε_0_ along the *a*, *b*, and *c* directions, respectively, using AMSET.^29^ Herein, the Kumagai–Oba (eFNV) finite-size charge correction scheme was applied due to the anisotropic character of Sb_2_O_5_.^52^

**Figure 4:**
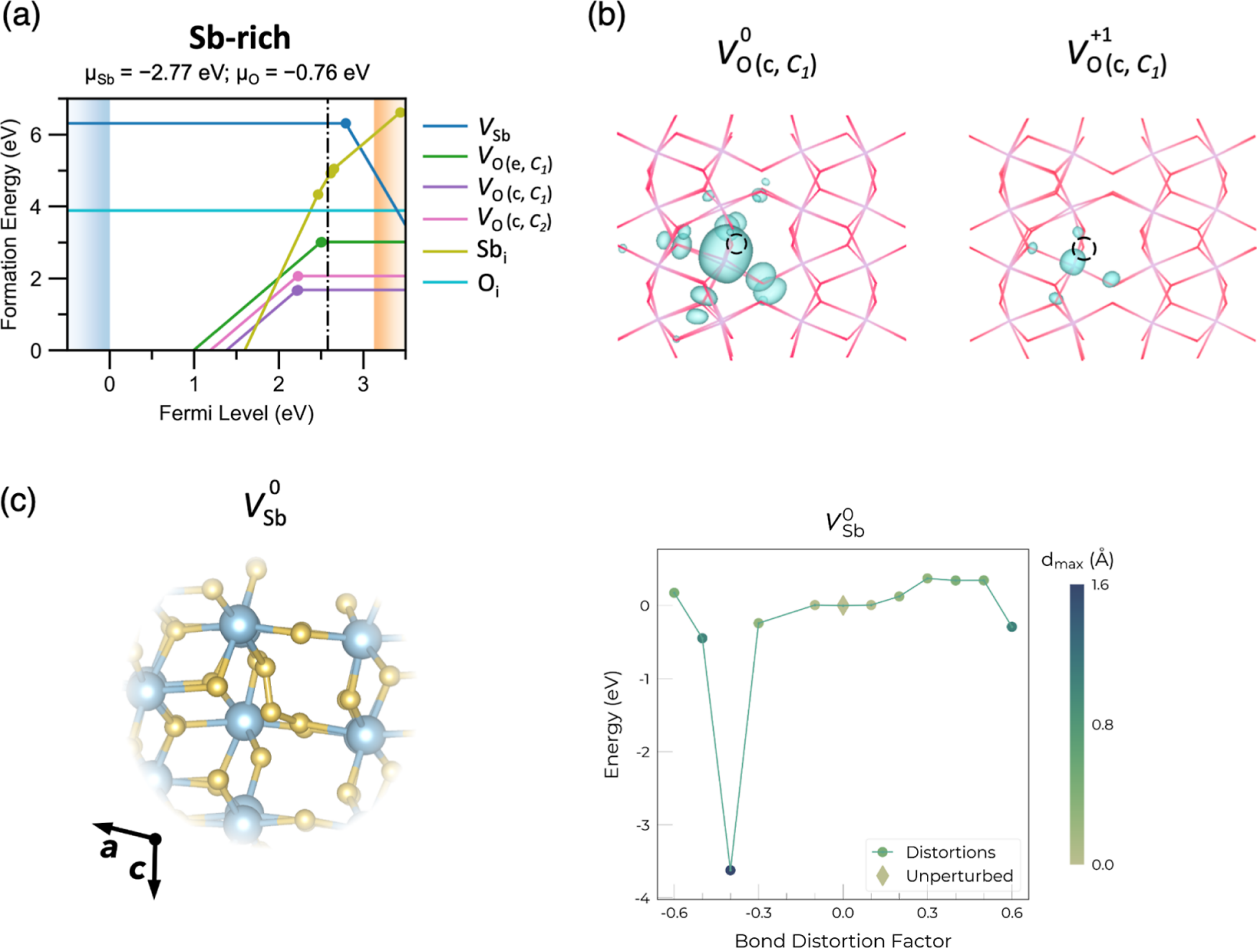
(a) Transition level diagram of intrinsic defects in Sb_2_O_5_ under Sb-rich and O-poor (n-type) conditions, with a self-consistent Fermi level at 2.58 eV above the VBM. (b) Charge density isosurfaces of the highest-occupied state of *V*_O(c,*C*_1_)_ in 0 and +1 charge states using a wireframe structure, plotted with an isosurface depth of 0.032 e Å^–3^ and viewed along the *c* direction. O atoms correspond to the intersection of red lines, Sb to the intersections of purple lines, and the black dotted circle indicates the oxygen vacancy. (c) Visualization of the O–O–O trimer formed in the ground-state structure of *V*_Sb_^0^, alongside the ShakeNBreak^33,34^ plot of energy versus initial bond distortion factor. Here, the ground-state structure is found with −0.4 (−40%) bond distortion.

As a binary semiconductor, Sb_2_O_5_ has six possible intrinsic point defects, namely, antimony interstitials (Sb_i_), oxygen interstitials (O_i_), antimony vacancies (*V*_Sb_), oxygen vacancies (*V*_O_), antimony-on-oxygen antisites (Sb_O_), and oxygen-on-antimony antisites (O_Sb_). However, the analyses of O_Sb_ and Sb_O_ are excluded as they are expected to be energetically unfavorable due to their large size and charge mismatch. Figure 4b shows the formation energy diagram for all intrinsic point defects under Sb-rich/O-poor conditions (i.e., the conditions most conducive to n-type behavior).

Three different *V*_O_ exist due to the three crystallographically distinct oxygen sites [one edge-sharing, , and two corner-sharing,  and ], each acting as deep donors and having the lowest formation energies among all intrinsic point defects—as is often the case in conventional TCOs such as In_2_O_3_, SnO_2_, and ZnSb_2_O_6_.^8,10,53^ Among the three oxygen vacancies,  exhibits negative-U behavior where the lowest energy charge state changes directly from +2 to neutral as the Fermi level moves toward the CBM.^54^ This is because the formation of a neutral oxygen vacancy creates two electrons that are mainly localized on the neighboring Sb s orbital, where it favors full or zero occupation over partial occupation. In addition, no major symmetry-breaking is identified for *V*_O_^+1^, with similar charge localization to the neutral vacancy state, as shown in Figure 4b, and thus, a negative-U behavior is preferred. The other two oxygen vacancies [ and ] have a narrow stability window of 15 and 10 meV for the +1 charge state, respectively. While oxygen vacancies are the lowest energy intrinsic defect, they still have significant formation energies, with *E*_f_() = 1.68 eV—suggesting that they will form in appreciable but not large concentrations unless very high processing temperatures are used. The charge density of the lowest formation energy  is shown in Figure 4b. Upon formation of the oxygen vacancy in its neutral state, two extra electrons are generated, which prefer to occupy the lowest energy unoccupied states—the Sb s orbitals of the CBM. This is seen in Figure 4b, where the electron densities associated with *V*_O_^0^ and *V*_O_^+1^ are mainly localized on the Sb atom neighboring the vacancy site. *V*_O_^+2^ is a fully ionized state where no additional electron/charge is present.

O_i_ is the next lowest energy defect, which has a formation energy of 3.89 eV under O-poor conditions (Figure 4). It is stable as a neutral dimer across all Fermi levels, with an O–O bond length of 1.40 Å. This is known as a peroxide species, which has also been found in other n-type oxides.^34,55−57^ Sb_i_ is a donor defect in Sb_2_O_5_, with (+5/+4), (+4/+3), and (+3/+2) transition levels located within the bandgap, where the transition level is defined as the position of the Fermi level when two different charge states of a defect have the same energy. Sb_i_ prefers to occupy octahedral sites, where it is surrounded by six O atoms, resembling the typical Sb–O coordination environment found in the bulk structure. For Sb_i_^+5^, the average Sb_i_–O bond length is 1.96 Å, similar to the bulk Sb–O bond length of 1.99 Å. *V*_Sb_ has much higher formation energy at 6.31 eV for *V*_Sb_^0^ under O-poor conditions, where *V*_Sb_^–4^ cuts the CBM at around 5.0 eV, and so is much less likely to form. High formation energies for *V*_Sb_ were also observed in ZnSb_2_O_6_^10^ and can be attributed to the high oxidation state (+5) of Sb in this compound. However, under Sb-poor conditions, the formation energy of *V*_Sb_ at the CBM drops by ∼2 to ∼3 eV, becoming one of the lowest energy native defects under these conditions. Notably, *V*_Sb_ here is found to be a rare four-electron negative-U center, where only the (0/–4) charge transition level occurs within the bandgap—indicating that the equilibrium charge state of antimony vacancies changes directly from neutral to −4 as the Fermi level approaches the CBM. Negative-U behavior is typically related to structural reconstruction and lattice distortion, and so the structures of *V*_Sb_^0^ and *V*_Sb_^–4^ were investigated in detail.^54^ As shown in Figure 4c, an O–O–O trimer is formed in *V*_Sb_^0^, similar to the Se–Se–Se trimer found for *V*_Sb_ in Sb_2_Se_3_, which also results in 4-electron negative-U behavior.^54^ The two O–O bonds have distinct bond lengths of 1.28 and 1.41 Å, matching those of ozone/superoxides (1.2–1.3 Å) and peroxides (1.4–1.5 Å), respectively.^58−60^ This trimer geometry was identified using ShakeNBreak where the energy was found to be around 4 eV lower than that of the structure obtained with a standard (unperturbed) defect geometry relaxation (Figure 4c). On the other hand, for *V*_Sb_ in +3, +4, and +5 charge states, we find a split-vacancy configuration to be the preferred arrangement (Figure S6). A split-vacancy configuration can be thought of as a divacancy and interstitial cluster as a neighboring host Sb atom displaces toward the vacancy position (creating an additional vacancy and interstitial in the process). This type of vacancy structure is often formed in cation vacancies with high charge states, having also been reported in α-Al_2_O_3_ and β-Ga_2_O_3_.^61,62^

The thermodynamic analysis of intrinsic point defects in Sb_2_O_5_ above demonstrates that the oxygen vacancies are the lowest formation energy defects. However, they are still moderately high in energy, and the donor levels are deep in the bandgap (0.9–0.6 eV below the CBM). Therefore, there will not be any significant intrinsic n-type doping in this system. However, Sb_2_O_5_ has a large n-type doping window of around 5.0 eV. The doping window is defined as the formation energy of the lowest energy-compensating defect species at the corresponding band edge as this sets an upper limit to the formation energy of dopants, which could push the Fermi level close to the band edge without being negated by ionic charge compensation from intrinsic defects. O_i_ does not charge-compensate as it stays neutral across the bandgap. In this case, the donor doping is charge-compensated by the native acceptor *V*_Sb_^–4^, cutting the CBM at around 5.0 eV under O-poor conditions. Compared with ZnSb_2_O_6_,^10^ Sb_2_O_5_ has a wider n-type doping window since the dominant intrinsic acceptor (cation vacancy *V*_Sb_) here has higher energy than that of *V*_Zn_ because of the higher oxidation state of Sb.

The self-consistent Fermi level under O-poor conditions was predicted using the PY-SC-FERMI package.^63,64^ An annealing/growth temperature of 700 °C was assumed as it is a widely reported growth temperature in the literature for Sb_2_O_5_ synthesis.^16,41,65^ To understand the defect behavior at operating (room) temperature, the “frozen defect approach” was applied where the total concentration of each defect is fixed to the equilibrium concentration at the annealing temperature. Upon cooling, the total concentration of each defect remains unchanged, whereas the relative populations of each defect charge state are allowed to re-equilibrate. The self-consistent Fermi level is then recalculated using the operating temperature (300 K). Figure 4b shows that the self-consistent Fermi level in undoped Sb_2_O_5_ sits at around 0.50 eV lower than the CBM at room temperature, corresponding to an essentially negligible electron concentration of around 10^8^ cm^–3^, and showing undoped Sb_2_O_5_ to be highly insulating as expected.

### Extrinsic Defect Chemistry

From the charge transport analysis earlier, Sb_2_O_5_ was found to have good electron mobility, but to achieve a high conductivity comparable to conventional TCOs, high carrier (and thus doping) concentrations are required. However, at high doping concentrations, the impact of extrinsic dopants on the host lattice and carrier scattering can severely diminish the carrier mobilities. Therefore, to optimize the electrical conductivity, the objective is to achieve electron-doping while minimizing disruption of the CBM states (in order to retain decent carrier mobilities). As shown in Figure 2, Sb 5s states mainly comprise the CBM, followed by the states of O 2p. Considering O is the minor contributor to the CBM, doping on the O site is preferred since it is likely to have a smaller impact on electron mobilities. In addition, the relatively small *V*_O_ but large *V*_Sb_ formation energies shown in the intrinsic transition level diagram (Figure 4b) also suggest that substituting on O will be easier as removing O from the lattice requires less energy than removing Sb. Fluorine (F) was selected as a potential dopant due to its similar ionic radius (1.29 Å) to that of oxygen (1.35 Å) and adjacent position to that of oxygen on the periodic table (having similar 2p valence orbital energies). Combined, these similarities are likely to yield low formation energies for F_O_ substitutional donors and thus high achievable electron concentrations. In other TCOs such as ZnO, SnO_2_, and TiO_2_, F has also often been incorporated as an O-site dopant to enhance performance.^66−68^

**Figure 5:**
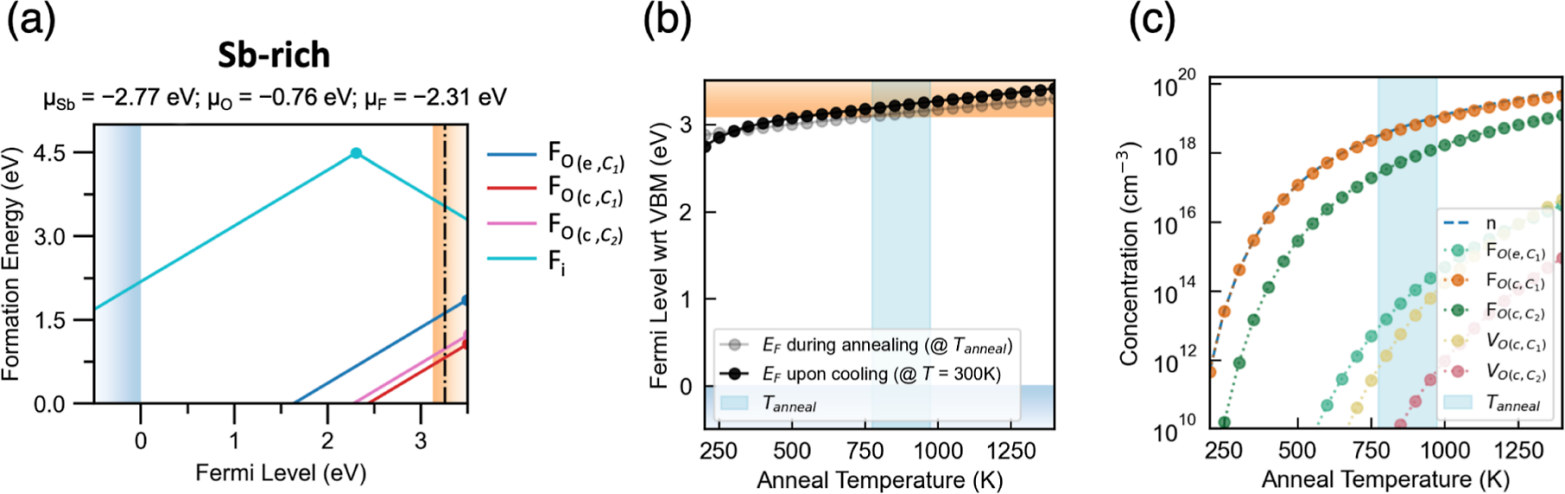
(a) Transition level diagram of low-energy extrinsic defects in F-doped Sb_2_O_5_ under Sb-rich and O-poor (n-type) conditions with the predicted self-consistent Fermi level located at 0.18 eV above the CBM. (b) Calculated self-consistent Fermi level positions in F-doped Sb_2_O_5_ during annealing (gray) and upon cooling to operating temperature (300 K; black). The light blue block shows a range of growth temperatures from 500 to 700 °C reported in the literature.^16,41,65,69^ (c) Room-temperature carrier and defect concentrations as a function of annealing temperature. Defects with concentrations lower than 1 × 10^10^ cm^–3^ are omitted from the legend and are plotted using DOPED and PY-SC-FERMI.^40,63,64^

Figure 5a shows the transition level diagram for the low-energy extrinsic point defects in F-doped Sb_2_O_5_ under Sb-rich/O-deficient conditions, in which the limiting secondary phase is Sb_2_F_7_. F_i_ is found to be a high-energy defect with a formation energy of around 3.7 eV in its negative charge state at the CBM, precluding its formation at any significant concentration within the material and thus having a negligible impact on the conductivity. By comparing F_i_ with the intrinsic O_i_ interstitial, it can be seen that F_i_ has a higher formation energy for most (n-type) Fermi levels. Usually, the smaller ionic radius of F would suggest F_i_ formation to be more favorable, especially in ionic compounds.^70^ However, here, O_i_ is more stable due to the formation of the O–O dimer, which stabilizes the neutral state. It is also noticeable that F_i_ is a negative-U center with F_i_^+1^ stability across a large range of Fermi levels, despite this being a somewhat unusual charge state from simple oxidation-state considerations. Similar negative-U behavior of F_i_^+1^ is observed in another n-type oxide, BaBi_2_O_6_.^71^ This defect species is stabilized by the formation of a short O–F_i_ bond with a corner-sharing oxygen atom, with a bond length (1.40 Å) similar to that in OF_2_ (1.41 Å), annihilating the two holes associated with this species.^72^

Fluorine-on-oxygen substitutions, on the other hand, have quite low formation energies with the most favorable substitution site [] having a formation energy of 0.60 eV in the +1 charge state at the CBM. In , the newly formed two Sb–F bonds have longer bond lengths (2.07 and 1.99 Å) than the two Sb–O bond lengths in bulk Sb_2_O_5_ (1.92 and 1.89 Å). The low formation energy of F_O_ indicates its ready solubility in Sb_2_O_5_, and is analogous to that observed in ZnSb_2_O_6_.^10^ All F_O_ substitutions are resonant defects where the (+1/0) transition level sits well within the conduction band, indicating that high carrier concentrations can be achieved through F doping. Among the three substitutional defects, the corner-sharing sites  and  have lower formation energies (0.60 and 0.77 eV at the CBM, respectively), reflecting the trend in oxygen vacancy formation energies (Figure 4a).

The calculated self-consistent Fermi levels and defect/electron concentrations upon F doping are presented as functions of the annealing temperature in Figure 5b,c. During the annealing process, a significant amount of  is formed, with its concentration equal to the overall electron concentrations at all temperatures due to  being the dominant contributor to the conductivity of F-doped Sb_2_O_5_. Due to the lack of low-energy-compensating acceptor species, the electron concentrations and Fermi levels under annealing and after cooling are mostly similar. The self-consistent Fermi level *E*_F_ at room temperature is predicted to lie at 0.18 eV above the conduction band, assuming an annealing temperature of 700 °C.^16,41,65^ The electron concentration at this predicted *E*_F_ reaches 10^19^ cm^–3^, which is around 1 order of magnitude higher than the Mott criterion. At this carrier concentration, the predicted mobility and conductivity of F-doped Sb_2_O_5_ are around 103 cm^2^ V^–1^ s^–1^ and 100 S cm^–1^, respectively, at room temperature, where the limiting scattering mechanism is mainly POP (Figure 3). In comparison to that of conventional n-type TCOs, this predicted conductivity for F-doped Sb_2_O_5_ is somewhat modest. If a greater conductivity is desired, a higher annealing temperature can be used, as demonstrated in Figure 5c, or potentially nonequilibrium growth conditions, where more fluorine can be incorporated to yield higher carrier concentrations. Moreover, the neglect of temperature-dependent renormalization of the host band gap may also contribute to a slight underestimation of the equilibrium electron concentrations under F-doping here. The calculation is also limited by the relatively simple model used in the AMSET package to address impurity scattering as the effect of a relatively significant F_O_ concentration on the lattice is not explicitly accounted for in the carrier mobility model. Overall, the low-energy formation of resonant F_O_ defects is predicted to push the self-consistent Fermi level into the conduction band, yielding a strongly degenerate semiconducting behavior upon F-doping in Sb_2_O_5_.

### Band Alignment

**Figure 6:**
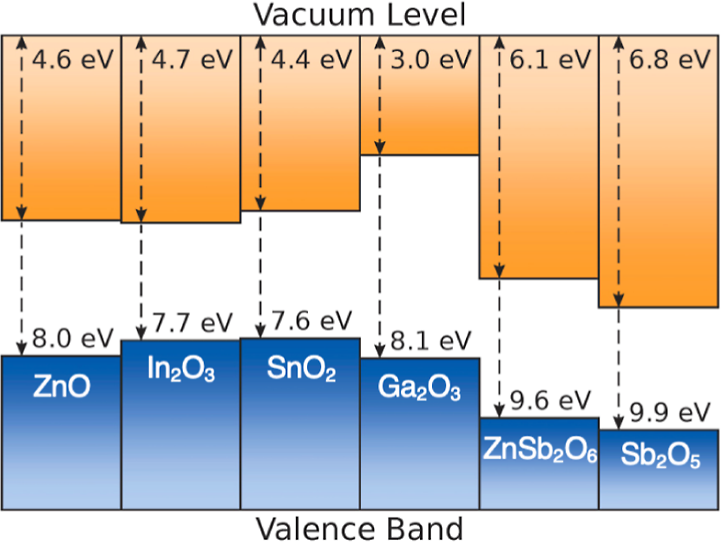
Electronic band alignment of Sb_2_O_5_ with existing TCOs. The values for Sb_2_O_5_, Ga_2_O_3_, and ZnSb_2_O_6_ are calculated from theory, while the others are experimental values.^7,10,73−75^.

The electronic band alignment of Sb_2_O_5_ was investigated and compared with that of existing TCOs, as shown in Figure 6. The ionization potential and the EA were calculated to be 9.89 and 6.80 eV, respectively. The EA of Sb_2_O_5_ is significantly larger than that of established TCOs. This is because the Sb 5s states mainly contribute to the CBM, as for ZnSb_2_O_6_ and similar to other TCOs where the cation *n*s orbitals comprise the CBM.^10^ However, the *n*s energy levels differ due to the effective nuclear charge and electron shielding effects. Other conventional post-transition metals have higher energy for the unoccupied *n*s states since they have lower oxidation states (+2/+3/+4) than Sb(V) and thus reduced nuclear attraction.^70^ Therefore, the Sb 5s orbitals in the +5 oxidation state have a smaller nuclear-electron distance, which has less shielding and stronger attraction, causing the Sb s states to sit at lower energy. Indeed, the fifth ionization energy of Sb is much higher than the fourth and third ionization energies of Sn and In.^76^ Moreover, this brings the Sb s orbital closer in energy to the deep 2p states, giving rise to a stronger interaction in the conduction band. The high oxidation state and smaller ionic radius of Sb^5+^ also result in a reduced Sb–O bond length compared to those of Sn–O and In–O, contributing to the strength of the Sb 5s–O 2p interactions. The resulting dispersed conduction band further extends the energy range of the unoccupied states, yielding a larger EA. This large EA could be particularly beneficial for TCO applications in organic solar cells, aiding the acceptance of photoexcited electrons from the organic active layer and thus facilitating electron extraction, reducing the likelihood of electron–hole recombination, and potentially boosting efficiencies.^77^ Sb_2_O_5_ with a high EA could therefore be a cheaper choice of TCO that can provide more diversity to the optoelectronics market.

## Conclusions

An investigation into the potential of Sb_2_O_5_ as an n-type TCO was conducted by using first-principles calculations. The analysis of its electronic structure revealed a highly dispersed conduction band, indicative of high electron mobility. A large optical band gap of around 3.6 eV was predicted, demonstrating visible-light transparency. Although undoped Sb_2_O_5_ was found to be insulating, a wide n-type doping window facilitates the introduction of extrinsic dopants, which can provide high charge carrier concentrations. Fluorine was chosen as a candidate substitutional dopant due to its similar size to oxygen and the low formation energies of oxygen vacancies. The predicted carrier concentration of F-doped Sb_2_O_5_ surpassed the Mott criterion, pushing the self-consistent Fermi level into the conduction band. The results show that F-doped Sb_2_O_5_ exhibits degenerate n-type transparent conducting behavior. Furthermore, the electronic band alignment demonstrates an extremely large EA in Sb_2_O_5_, enhancing its suitability in organic solar cells, where the overall efficiency can be improved along with a lower cost. These findings corroborate the potential of Sb(V)-based oxides as alternative earth-abundant n-type TCOs, providing much-needed diversity in the field of transparent conducting materials.

## Supporting Information Available

Calculated lattice parameters, phonon dispersion, Tauc Plot, COHP analysis, data for competing phase formation energies for calculating the chemical potential limits of both intrinsic and extrinsic defects, thermostability region plot, intrinsic and extrinsic transition level diagrams under p-type (Sb-poor and O-rich) conditions, visualization of *V*_Sb_^–4^ split-vacancy configuration, and all the energy terms in eq 1 used for calculating the defect formation energy (PDF)

## References

[ref1] Willis J.; Scanlon D. O. Latest directions in p-type transparent conductor design. J. Mater. Chem. C 2021, 9, 11995–12009. 10.1039/D1TC02547C.

[ref2] Brunin G.; Ricci F.; Ha V.-A.; Rignanese G.-M.; Hautier G. Transparent conducting materials discovery using high-throughput computing. npj Comput. Mater. 2019, 5, 63 10.1038/s41524-019-0200-5.

[ref3] Bädeker K. Über die elektrische Leitfähigkeit und die thermoelektrische Kraft einiger Schwermetallverbindungen. Ann. Phys. (Berlin, Ger.) 1907, 327, 749–766. 10.1002/andp.19073270409.

[ref4] Grundmann M. Karl Bädeker (1877–1914) and the discovery of transparent conductive materials: Karl Bädeker. Phys. Status Solidi A 2015, 212, 1409–1426. 10.1002/pssa.201431921.

[ref5] Ueda N.; Maeda H.; Hosono H.; Kawazoe H. Band-gap widening of CdO thin films. J. Appl. Phys. 1998, 84, 6174–6177. 10.1063/1.368933.

[ref6] Granqvist C.; Hultåker A. Transparent and conducting ITO films: new developments and applications. Thin Solid Films 2002, 411, 1–5. 10.1016/S0040-6090(02)00163-3.

[ref7] Zhang J.; Willis J.; Yang Z.; Lian X.; Chen W.; Wang L.-S.; Xu X.; Lee T.-L.; Chen L.; Scanlon D. O.; Zhang K. H. Deep UV transparent conductive oxide thin films realized through degenerately doped wide-bandgap gallium oxide. Cell Rep. Phys. Sci. 2022, 3, 100801 10.1016/j.xcrp.2022.100801.

[ref8] Williamson B. A. D.; Featherstone T. J.; Sathasivam S. S.; Swallow J. E. N.; Shiel H.; Jones L. A. H.; Smiles M. J.; Regoutz A.; Lee T. L.; Xia X.; et al. Resonant Ta Doping for Enhanced Mobility in Transparent Conducting SnO_2_. Chem. Mater. 2020, 32, 1964–1973. 10.1021/acs.chemmater.9b04845. 32296264 PMC7147269

[ref9] Bamiduro O.; Mustafa H.; Mundle R.; Konda R. B.; Pradhan A. K. Metal-like conductivity in transparent Al:ZnO films. Appl. Phys. Lett. 2007, 90, 252108 10.1063/1.2749836.

[ref10] Jackson A. J.; Parrett B. J.; Willis J.; Ganose A. M.; Leung W. W. W.; Liu Y.; Williamson B. A. D.; Kim T. K.; Hoesch M.; Veiga L. S. I.; et al. Computational Prediction and Experimental Realization of Earth-Abundant Transparent Conducting Oxide Ga-Doped ZnSb_2_O_6_. ACS Energy Lett. 2022, 7, 3807–3816. 10.1021/acsenergylett.2c01961. 36398093 PMC9664443

[ref11] Hautier G.; Miglio A.; Waroquiers D.; Rignanese G.-M.; Gonze X. How Does Chemistry Influence Electron Effective Mass in Oxides? A High-Throughput Computational Analysis. Chem. Mater. 2014, 26, 5447–5458. 10.1021/cm404079a.

[ref12] Kikuchi N.; Hosono H.; Kawazoe H.; Tanegashima O.; Ota I.; Kimura Y. Carrier Generation in Wide-Gap Conductor, Zinc Antimonate. J. Am. Ceram. Soc. 2005, 88, 2793–2797. 10.1111/j.1551-2916.2005.00528.x.

[ref13] Tamaki J.; Yamada Y.; Yamamoto Y.; Matsuoka M.; Ota I. Sensing properties to dilute hydrogen sulfide of ZnSb_2_O_6_ thick-film prepared by dip-coating method. Sens. Actuators, B 2000, 66, 70–73. 10.1016/S0925-4005(99)00408-6.

[ref14] Zhu B.; Xie C.; Wang A.; Zeng D.; Hu M.; Wang W. Electrical conductivity and gas sensitivity of Zn–Sb–O thick films. Mater. Res. Bull. 2004, 39, 409–415. 10.1016/j.materresbull.2003.10.011.

[ref15] U.S. Geological Survey. Mineral Commodity Summaries 2023, 2023, p 210.

[ref16] Jansen M. Crystal Structure of Sb_2_O_5_. Angew. Chem., Int. Ed. Engl. 1978, 17, 137 10.1002/anie.197801371.

[ref17] Allen J. P.; Carey J. J.; Walsh A.; Scanlon D. O.; Watson G. W. Electronic Structures of Antimony Oxides. J. Phys. Chem. C 2013, 117, 14759–14769. 10.1021/jp4026249.

[ref18] Kresse G.; Hafner J. Ab initio molecular dynamics for liquid metals. Phys. Rev. B 1993, 47, 558–561. 10.1103/PhysRevB.47.558. 10004490

[ref19] Kresse G.; Furthmüller J. Efficient iterative schemes for ab initio total-energy calculations using a plane-wave basis set. Phys. Rev. B: Condens. Matter Mater. Phys. 1996, 54, 11169–11186. 10.1103/PhysRevB.54.11169. 9984901

[ref20] Kresse G.; Hafner J. Ab initio molecular-dynamics simulation of the liquid-metal–amorphous-semiconductor transition in germanium. Phys. Rev. B: Condens. Matter Mater. Phys. 1994, 49, 14251–14269. 10.1103/PhysRevB.49.14251. 10010505

[ref21] Kresse G.; Furthmüller J. Efficiency of ab-initio total energy calculations for metals and semiconductors using a plane-wave basis set. Comput. Mater. Sci. 1996, 6, 15–50. 10.1016/0927-0256(96)00008-0. 9984901

[ref22] Kresse G.; Joubert D. From ultrasoft pseudopotentials to the projector augmented-wave method. Phys. Rev. B: Condens. Matter Mater. Phys. 1999, 59, 1758–1775. 10.1103/PhysRevB.59.1758.

[ref23] Kresse G.; Hafner J. Norm-conserving and ultrasoft pseudopotentials for first-row and transition elements. J. Phys.: Condens. Matter 1994, 6, 8245–8257. 10.1088/0953-8984/6/40/015.

[ref24] Perdew J. P.; Ruzsinszky A.; Csonka G. I.; Vydrov O. A.; Scuseria G. E.; Constantin L. A.; Zhou X.; Burke K. Restoring the Density-Gradient Expansion for Exchange in Solids and Surfaces. Phys. Rev. Lett. 2008, 100, 136406 10.1103/PhysRevLett.100.136406. 18517979

[ref25] Adamo C.; Barone V. Toward reliable density functional methods without adjustable parameters: The PBE0 model. J. Chem. Phys. 1999, 110, 6158–6170. 10.1063/1.478522.

[ref26] Kavanagh S. R. vaspup 2.0. 2023. https://doi.org/10.5281/zenodo.8408542.

[ref27] Momma K.; Izumi F. VESTA: a three-dimensional visualization system for electronic and structural analysis. J. Appl. Crystallogr. 2008, 41, 653–658. 10.1107/S0021889808012016.

[ref28] M Ganose A.; J Jackson A.; O Scanlon D. sumo: Command-line tools for plotting and analysis of periodic ab initio calculations. J. Open Source Software 2018, 3, 717 10.21105/joss.00717.

[ref29] Ganose A. M.; Park J.; Faghaninia A.; Woods-Robinson R.; Persson K. A.; Jain A. Efficient calculation of carrier scattering rates from first principles. Nat. Commun. 2021, 12, 2222 10.1038/s41467-021-22440-5. 33850113 PMC8044096

[ref30] Spooner K.; Einhorn M.; Davies D. W.; Scanlon D. O. ThermoParser: streamlined analysis of thermoelectric properties. 2023. https://github.com/SMTG-Bham/ThermoParser (accessed Oct 05, 2023).

[ref31] Togo A.; Tanaka I. First principles phonon calculations in materials science. Scr. Mater. 2015, 108, 1–5. 10.1016/j.scriptamat.2015.07.021.

[ref32] Brlec K.; Davies D.; Scanlon D. Surfaxe: Systematic surface calculations. J. Open Source Software 2021, 6, 3171 10.21105/joss.03171.

[ref33] Mosquera-Lois I.; Kavanagh S. R.; Walsh A.; Scanlon D. O. ShakeNBreak: Navigating the defect configurational landscape. J. Open Source Software 2022, 7, 4817 10.21105/joss.04817.

[ref34] Mosquera-Lois I.; Kavanagh S. R.; Walsh A.; Scanlon D. O. Identifying the ground state structures of point defects in solids. npj Comput. Mater. 2023, 9, 25 10.1038/s41524-023-00973-1.

[ref35] Scanlon D. O.; Kehoe A. B.; Watson G. W.; Jones M. O.; David W. I. F.; Payne D. J.; Egdell R. G.; Edwards P. P.; Walsh A. Nature of the Band Gap and Origin of the Conductivity of PbO_2_ Revealed by Theory and Experiment. Phys. Rev. Lett. 2011, 107, 246402 10.1103/PhysRevLett.107.246402. 22243014

[ref36] Lany S.; Zunger A. Assessment of correction methods for the band-gap problem and for finite-size effects in supercell defect calculations: Case studies for ZnO and GaAs. Phys. Rev. B: Condens. Matter Mater. Phys. 2008, 78, 235104 10.1103/PhysRevB.78.235104.

[ref37] Lany S.; Zunger A. Accurate prediction of defect properties in density functional supercell calculations. Modell. Simul. Mater. Sci. Eng. 2009, 17, 084002 10.1088/0965-0393/17/8/084002.

[ref38] Makov G.; Payne M. C. Periodic boundary conditions in ab initio calculations. Phys. Rev. B: Condens. Matter Mater. Phys. 1995, 51, 4014–4022. 10.1103/PhysRevB.51.4014. 9979237

[ref39] Murphy S. T.; Hine N. D. M. Anisotropic charge screening and supercell size convergence of defect formation energies. Phys. Rev. B: Condens. Matter Mater. Phys. 2013, 87, 094111 10.1103/PhysRevB.87.094111.

[ref40] Kavanagh S. R. Doped. https://doi.org/10.21105/joss.06433.

[ref41] Mindil A.; Mohamed S. H.; Amri N.; Rabia M. Morphological and optical characterizations of Sb_2_O_5_ nanorods as new photoelectrode for hydrogen generation. Phys. Scr. 2023, 98, 105907 10.1088/1402-4896/acf351.

[ref42] Walsh A.; Da Silva J. L. F.; Wei S.-H.; Körber C.; Klein A.; Piper L. F. J.; DeMasi A.; Smith K. E.; Panaccione G.; Torelli P.; Payne D. J.; Bourlange A.; Egdell R. G. Nature of the Band Gap of In_2_O_3_ Revealed by First-Principles Calculations and X-Ray Spectroscopy. Phys. Rev. Lett. 2008, 100, 167402 10.1103/PhysRevLett.100.167402. 18518246

[ref43] Kavanagh S. R.; Savory C. N.; Scanlon D. O.; Walsh A. Hidden spontaneous polarisation in the chalcohalide photovoltaic absorber Sn_2_SbS_2_I_3_. Mater. Horiz. 2021, 8, 2709–2716. 10.1039/D1MH00764E. 34617541 PMC8489399

[ref44] Edwards P. P.; Sienko M. J. Universality aspects of the metal-nonmetal transition in condensed media. Phys. Rev. B: Condens. Matter Mater. Phys. 1978, 17, 2575–2581. 10.1103/PhysRevB.17.2575.

[ref45] Mott N. F. Metal-Insulator Transition. Rev. Mod. Phys. 1968, 40, 677–683. 10.1103/RevModPhys.40.677.

[ref46] Pergament A. Metal–insulator transition: the Mott criterion and coherence length. J. Phys.: Condens. Matter 2003, 15, 3217–3223. 10.1088/0953-8984/15/19/322.

[ref47] Feneberg M.; Nixdorf J.; Lidig C.; Goldhahn R.; Galazka Z.; Bierwagen O.; Speck J. S. Many-electron effects on the dielectric function of cubic In_2_O_3_: Effective electron mass, band nonparabolicity, band gap renormalization, and Burstein-Moss shift. Phys. Rev. B 2016, 93, 045203 10.1103/PhysRevB.93.045203.

[ref48] Serin T.; Yildiz A.; Serin N.; Yildirim N.; Özyurt F.; Kasap M. Electron–Electron Interactions in Sb-Doped SnO_2_ Thin Films. J. Electron. Mater. 2010, 39, 1152–1158. 10.1007/s11664-010-1252-y.

[ref49] Kuang Y.; Ma T. C.; Chen X. H.; Li J.; Ren F.-F.; Liu B.; Cui X. Y.; Ringer S. P.; Zhu S. M.; Gu S. L.; Zhang R.; Zheng Y. D.; Ye J. D. Misfit epitaxial strain manipulated transport properties in cubic In_2_O_3_ hetero-epilayers. Appl. Phys. Lett. 2020, 117, 102104 10.1063/5.0021344.

[ref50] Fiedler A.; Ramsteiner M.; Galazka Z.; Irmscher K. Raman scattering in heavily donor doped β-Ga_2_O_3_. Appl. Phys. Lett. 2020, 117, 152107 10.1063/5.0024494.

[ref51] Dixon S. C.; Scanlon D. O.; Carmalt C. J.; Parkin I. P. n-Type doped transparent conducting binary oxides: an overview. J. Mater. Chem. C 2016, 4, 6946–6961. 10.1039/C6TC01881E.

[ref52] Kumagai Y.; Oba F. Electrostatics-based finite-size corrections for first-principles point defect calculations. Phys. Rev. B: Condens. Matter Mater. Phys. 2014, 89, 195205 10.1103/PhysRevB.89.195205.

[ref53] Swallow J. E. N.; Williamson B. A. D.; Sathasivam S.; Birkett M.; Featherstone T. J.; Murgatroyd P. A. E.; Edwards H. J.; Lebens-Higgins Z. W.; Duncan D. A.; Farnworth M.; et al. Resonant doping for high mobility transparent conductors: the case of Mo-doped In_2_O_3_. Mater. Horiz. 2020, 7, 236–243. 10.1039/C9MH01014A.

[ref54] Wang X.; Kavanagh S. R.; Scanlon D. O.; Walsh A. Four-electron negative-U vacancy defects in antimony selenide. Phys. Rev. B 2023, 108, 134102 10.1103/PhysRevB.108.134102.

[ref55] Scanlon D. O. Defect engineering of BaSnO_3_ for high-performance transparent conducting oxide applications. Phys. Rev. B: Condens. Matter Mater. Phys. 2013, 87, 161201 10.1103/PhysRevB.87.161201.

[ref56] Scanlon D. O.; Watson G. W. On the possibility of p-type SnO_2_. J. Mater. Chem. 2012, 22, 25236 10.1039/c2jm34352e.

[ref57] Cen J.; Zhu B.; Kavanagh S. R.; Squires A. G.; Scanlon D. O. Cation disorder dominates the defect chemistry of high-voltage LiMn_1.5_Ni_0.5_O_4_ (LMNO) spinel cathodes. J. Mater. Chem. A 2023, 11, 13353–13370. 10.1039/D3TA00532A.

[ref58] Miliordos E.; Xantheas S. S. On the Bonding Nature of Ozone (O_3_) and Its Sulfur-Substituted Analogues SO_2_, OS_2_, and S_3_: Correlation between Their Biradical Character and Molecular Properties. J. Am. Chem. Soc. 2014, 136, 2808–2817. 10.1021/ja410726u. 24499187

[ref59] Lysenko K. A.; Antipin M. Y.; Khrustalev V. N. The nature of the O-O bond in hydroperoxides. Russ. Chem. Bull. 2001, 50, 1539–1549. 10.1023/A:1013013930181.

[ref60] Cramer C. J.; Tolman W. B.; Theopold K. H.; Rheingold A. L. Variable character of O-O and M-O bonding in side-on (η^2^) 1:1 metal complexes of O_2_. Proc. Natl. Acad. Sci. U.S.A. 2003, 100, 3635–3640. 10.1073/pnas.0535926100. 12634422 PMC152974

[ref61] Kononov A.; Lee C.-W.; Shapera E. P.; Schleife A. Identifying native point defect configurations in α -alumina. J. Phys.: Condens. Matter 2023, 35, 334002 10.1088/1361-648X/acd3cf. 37199124

[ref62] Varley J. B.; Peelaers H.; Janotti A.; Van De Walle C. G. Hydrogenated cation vacancies in semiconducting oxides. J. Phys.: Condens. Matter 2011, 23, 334212 10.1088/0953-8984/23/33/334212. 21813965

[ref63] Buckeridge J. Equilibrium point defect and charge carrier concentrations in a material determined through calculation of the self-consistent Fermi energy. Comput. Phys. Commun. 2019, 244, 329–342. 10.1016/j.cpc.2019.06.017.

[ref64] Squires A. G.; Scanlon D. O.; Morgan B. J. py-sc-fermi: self-consistent Fermi energies and defectconcentrations from electronic structure calculations. J. Open Source Software 2023, 8, 4962 10.21105/joss.04962.

[ref65] Kim S. S.; Na H. G.; Kwon Y. J.; Cho H. Y.; Kim H. W. Synthesis and room-temperature NO_2_ sensing properties of Sb_2_O_5_ nanowires. Met. Mater. Int. 2015, 21, 415–421. 10.1007/s12540-015-4264-6.

[ref66] Pham A. T. T.; Ngo N. M.; Le O. K. T.; Hoang D. V.; Nguyen T. H.; Phan T. B.; Tran V. C. High-mobility sputtered F-doped ZnO films as good-performance transparent-electrode layers. J. Sci.: Adv. Mater. Devices 2021, 6, 446–452. 10.1016/j.jsamd.2021.05.004.

[ref67] Supothina S.; De Guire M. R. Characterization of SnO_2_ thin films grown from aqueous solutions. Thin Solid Films 2000, 371, 1–9. 10.1016/S0040-6090(00)00989-5.

[ref68] Kafizas A.; Noor N.; Carmichael P.; Scanlon D. O.; Carmalt C. J.; Parkin I. P. Combinatorial Atmospheric Pressure Chemical Vapor Deposition of F:TiO_2_ ; the Relationship between Photocatalysis and Transparent Conducting Oxide Properties. Adv. Funct. Mater. 2014, 24, 1758–1771. 10.1002/adfm.201301333.

[ref69] Xiong H.-M.; Chen J.-S.; Li D.-M. Controlled growth of Sb_2_O_5_ nanoparticles and their use as polymer electrolyte fillers. J. Mater. Chem. 2003, 13, 1994–1998. 10.1039/B304342H.

[ref70] Shannon R. Revised effective ionic radii and systematic studies of interatomic distances in halides and chalcogenides. Acta Crystallogr. 1976, 32, 751–767. 10.1107/S0567739476001551.

[ref71] Spooner K. B.; Ganose A. M.; Leung W. W. W.; Buckeridge J.; Williamson B. A. D.; Palgrave R. G.; Scanlon D. O. BaBi_2_O_6_: A Promising n-Type Thermoelectric Oxide with the PbSb_2_O_6_ Crystal Structure. Chem. Mater. 2021, 33, 7441–7456. 10.1021/acs.chemmater.1c02164.

[ref72] Politzer P.; Habibollahzadeh D. Relationship between dissociation energies, force constants, and bond lengths for some N–F and O–F bonds. J. Chem. Phys. 1993, 98, 7659–7660. 10.1063/1.464679.

[ref73] Göpel W.; Brillson L. J.; Brucker C. F. Surface point defects and Schottky barrier formation on ZnO(1010). J. Vac. Sci. Technol. 1980, 17, 894–898. 10.1116/1.570612.

[ref74] Gassenbauer Y.; Klein A. Electronic and Chemical Properties of Tin-Doped Indium Oxide (ITO) Surfaces and ITO/ZnPc Interfaces Studied In-situ by Photoelectron Spectroscopy. J. Phys. Chem. B 2006, 110, 4793–4801. 10.1021/jp056640b. 16526716

[ref75] Helander M. G.; Greiner M. T.; Wang Z. B.; Tang W. M.; Lu Z. H. Work function of fluorine doped tin oxide. J. Vac. Sci. Technol., A 2011, 29, 011019 10.1116/1.3525641.

[ref76] Lide D. R. CRC Handbook of Chemistry and Physics, 84th ed.; CRC Press: Boca Raton, FL, 2003.

[ref77] Greiner M. T.; Lu Z.-H. Thin-film metal oxides in organic semiconductor devices: their electronic structures, work functions and interfaces. NPG Asia Mater. 2013, 5, e55 10.1038/am.2013.29.

