# Supplementary material for: Computational Prediction of an Antimony-Based n-Type Transparent Conducting Oxide: F-Doped Sb2O5
Source: Chem Mater. 2024 Mar 11;36(6):2907–16. doi: 10.1021/acs.chemmater.3c03257 (PMC10976629; doi:10.1021/acs.chemmater.3c03257)
Supplement: Supplementary file 1 — cm3c03257_si_001.pdf [file cm3c03257_si_001.pdf]

Computational Prediction of an Antimony-based n-type Transparent Conducting Oxide F-doped Sb_2O_5

Ke Li,[†,‡] Joe Willis,[†,‡] Seán R. Kavanagh,[†,‡,¶] and David O. Scanlon[*,§]

†*Department of Chemistry, University College London, 20 Gordon St, London, WC1H 0AJ, UK*

‡*Thomas Young Centre, University College London, Gower St, London, WC1E 6BT, UK*

¶*Department of Materials, Imperial College London, Exhibition Road, London, SW7 2AZ, UK*

§*School of Chemistry, University of Birmingham, Edgbaston, Birmingham, B15 2TT, UK*

E-mail: d.o.scanlon@bham.ac.uk

Phone: +44 (0)20 7679 4558

Supplementary Information

Crystal Structure

Table S1: Comparison of the formation energies of all Sb_2O_5 polymorphs identified in the Materials Project (MP) and ICSD databases.

Polymorphs	Space Group	Energy per Atom (eV/atom)
Sb_2O_5	*C2/c*	-8.31
Sb_2O_5	*Cmcm*	-8.26
Sb_2O_5	*Pbam*	-8.09
Sb_2O_5	*Pmmn*	-8.26
Sb_2O_5	$Fd\bar{3}m1$ (MP)	-6.74
Sb_2O_5	$Fd\bar{3}m1$ (ICSD)	-6.62

Table S2: Comparison of the conventional crystal lattice parameters for *C2/c* Sb_2O_5 relaxed by PBEsol, HSE06 and PBE0 DFT functional and the experimental values.[1]

Parameters	PBEsol	HSE06	PBE0	Experiment[1]
a / Å	12.72	12.64	12.63	12.65
b / Å	4.82	4.79	4.78	4.78
c / Å	5.47	5.43	5.43	5.42
β / °	103.73	103.78	103.78	103.93
Volume / $Å^3$	325.53	318.89	318.23	318.35

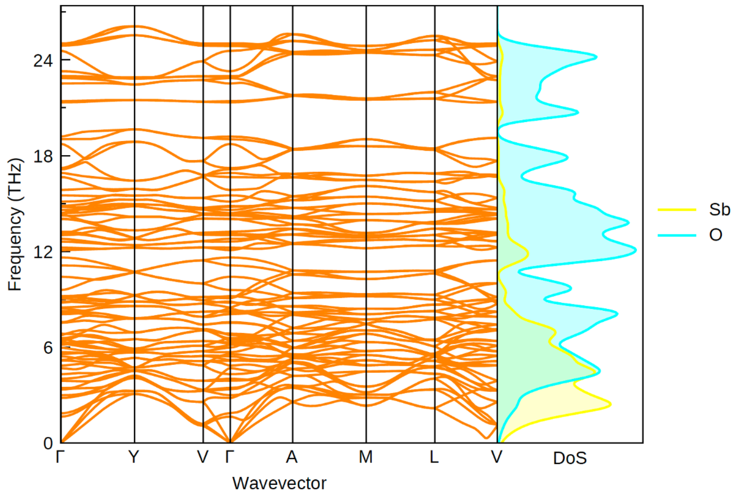

Figure S1: Phonon dispersion curve of Sb_2O_5 with projected density of states, calculated using a $1 \times 3 \times 3$ supercell.

The phonon dispersion of Sb_2O_5 was calculated using PBEsol with a $1 \times 3 \times 3$ supercell of the primitive unit cell. Figure S1 shows the phonon dispersion curve along the high-symmetry path in the Brillouin zone. No imaginary modes are observed in the phonon dispersion, indicating that Sb_2O_5 is dynamically stable at 0 K.

Electronic Structure

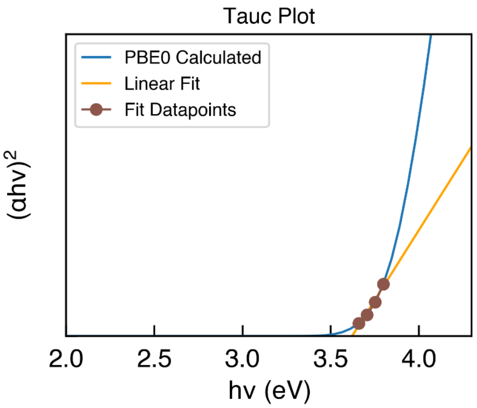

Figure S2: Direct gap Tauc fit of the calculated band-to-band optical absorption of Sb_2O_5, indicating an optical bandgap of $\sim$3.6 eV as determined by the intersection point of the linear fit.

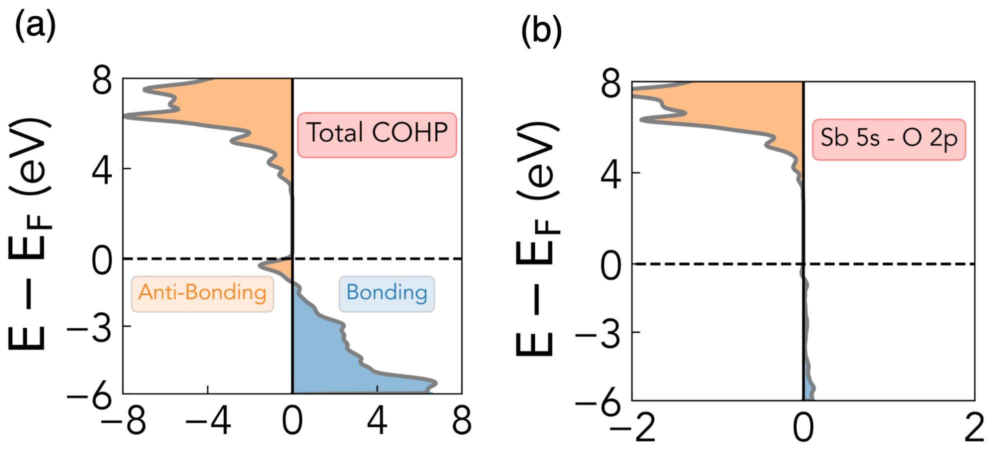

Figure S3: (a) Crystal Orbital Hamilton Population (COHP) analysis of the electronic density of states in Sb_2O_5 using LOBSTER, where the orange region indicates anti-bonding interaction and blue region indicates bonding character.[2–4] Fermi level set to the VBM. (b) COHP analysis of the predominantly anti-bonding interaction of Sb 5s and O 2p.[2–4]

Defect Chemistry

Table S3: Converged k-point mesh and the total energy per atom for competing phases for intrinsic defect study.

Species	k-point mesh	Formation energy / eV
Sb_2O_3	4 × 3 × 2	-7.82
SbO_2	4 × 4 × 2	-4.28
Sb_2O_5	6 × 6 × 5	-9.32

Table S4: Converged k-point mesh and the elemental energies.

Species	k-point mesh	Energy per atom / eV
Sb	9 × 9 × 9	-5.63
O	1 × 1 × 1	-7.52
F	1 × 1 × 1	-3.59

Table S5: Chemical potential limits for Sb_2O_5.

Conditions	μ_{Sb}	μ_O
Sb-poor/O-rich	-4.66	0
Sb-rich/O-poor	-2.77	-0.76

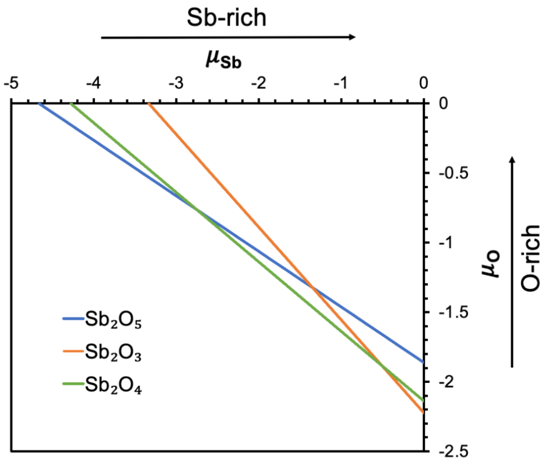

Figure S4: Thermostability regions of Sb_2O_5 and its competing phases in the Sb-O chemical space.

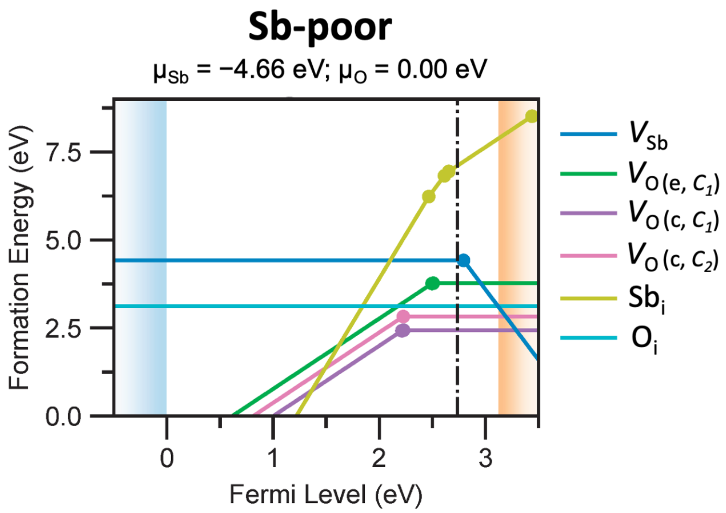

Figure S5: Transition level diagram of intrinsic defects in Sb_2O_5 under Sb-poor and O-rich (p-type) conditions.

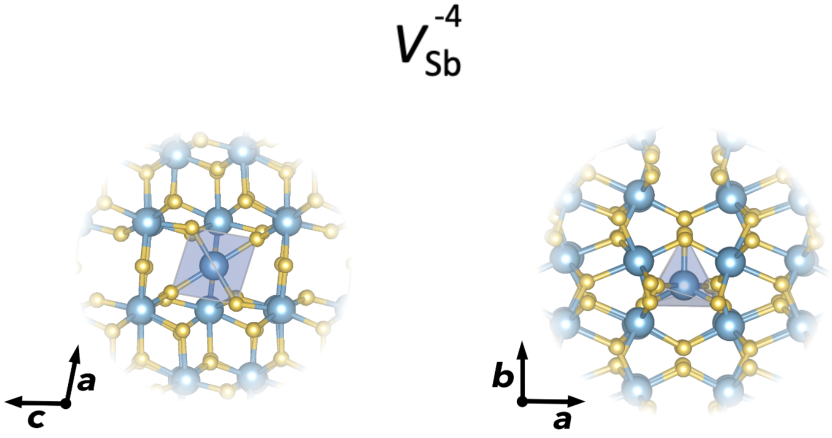

Figure S6: Visualization of the split-vacancy observed in V_{Sb}^{-4} along different directions.

Table S6: Converged k-point mesh and the total energy per atom for competing phases for extrinsic defect study. (*: different polymorphs.)

Competing phases	k-point mesh	Formation energy (eV)
Sb_2F_7	2 × 4 × 2	-21.69
Sb_4F_{15}	2 × 1 × 1	-45.63
Sb_7F_{29}	1 × 2 × 1	-85.67
$Sb_{11}F_{43}$	2 × 1 × 1	-129.49
SbF_3	4 × 4 × 2	-9.66
SbF_4	1 × 2 × 1	-11.99
SbF_5	1 × 3 × 2	-13.84
SbF_5*	4 × 4 × 2	-13.82
SbO_2F	4 × 4 × 3	-6.47
SbO_2F*	4 × 4 × 4	-6.41
$SbOF_3$	3 × 2 × 2	-10.19

Table S7: Calculated chemical potential limits under two different conditions for extrinsic defect study.

Conditions	μ_{Sb}	μ_O	μ_F	F limiting phase
Sb-rich/O-poor	-2.77	-0.76	-2.31	Sb_2F_7
Sb-poor/O-rich	-4.66	0.00	-1.84	$SbOF_3$

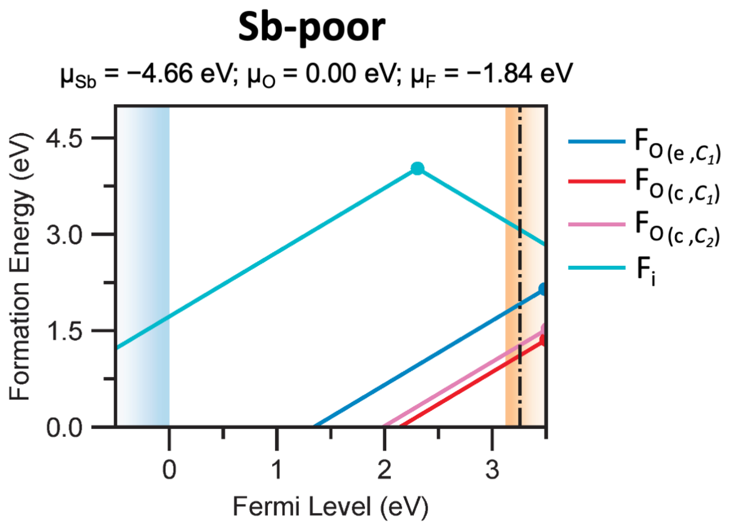

Figure S7: Transition level diagram of low energy extrinsic defects in F-doped Sb_2O_5 under Sb-poor and O-rich (p-type) conditions.

Table S8: The DOPED-generated intrinsic point defect formation energy table showing all the energy terms in Equation 1 used to calculate the defect formation energy (ΔE^{form}), where q is the defect charge state, ΔE^{raw} is the raw energy difference between defective supercell and bulk supercell, $q\epsilon_{VBM}$ is the product of defect charge states times the VBM eigenvalue, qE_F is the product of defect charge states times the Fermi level referenced to the VBM if qE_F is non-zero, $\Sigma\mu_{ref}$ is the sum of all reference energies of the elemental phases, $\Sigma\mu_{formal}$ is the sum of formal atomic chemical potential terms and E_{corr} is the finite-size supercell charge correction.[5]

Defect	q	ΔE^{raw}	$q\epsilon_{VBM}$	qE_F	$\Sigma\mu_{ref}$	$\Sigma\mu_{formal}$	E_{corr}	ΔE^{form}
O_i^0	0	-4.397	0	0	7.523	0.758	0	3.885
O_i^{-1}	-1	3.511	-4	0	7.523	0.758	-0.003	7.789
O_i^{-2}	-2	11.202	-8	0	7.523	0.758	0.083	11.566
Sb_i^{+5}	5	-40.961	20.001	0	5.626	2.766	4.434	-8.134
Sb_i^{+4}	4	-33.241	16.001	0	5.626	2.766	3.23	-5.618
Sb_i^{+3}	3	-25.524	12.001	0	5.626	2.766	2.162	-2.969
Sb_i^{+2}	2	-17.89	8	0	5.626	2.766	1.196	-0.302
Sb_i^{+1}	1	-9.754	4	0	5.626	2.766	0.53	3.168
Sb_i^0	0	-0.937	0	0	5.626	2.766	0	7.455

${V_{\mathrm{O(c,}\, C_1)}}^{+2}$	2	-2.922	8	0	-7.523	-0.758	0.438	-2.766
${V_{\mathrm{O(c,}\, C_1)}}^{+1}$	1	3.637	4	0	-7.523	-0.758	0.093	-0.551
${V_{\mathrm{O(c,}\, C_1)}}^{0}$	0	9.961	0	0	-7.523	-0.758	0	1.68
${V_{\mathrm{O(c,}\, C_2)}}^{+2}$	2	-2.516	8	0	-7.523	-0.758	0.389	-2.408
${V_{\mathrm{O(c,}\, C_2)}}^{+1}$	1	4.241	4	0	-7.523	-0.758	0.084	0.043
${V_{\mathrm{O(c,}\, C_2)}}^{0}$	0	10.349	0	0	-7.523	-0.758	0	2.067
${V_{\mathrm{O(e,}\, C_1)}}^{+2}$	2	-2.15	8	0	-7.523	-0.758	0.438	-1.993
${V_{\mathrm{O(e,}\, C_1)}}^{+1}$	1	4.696	4	0	-7.523	-0.758	0.092	0.507
${V_{\mathrm{O(e,}\, C_1)}}^{0}$	0	11.296	0	0	-7.523	-0.758	0	3.015
${V_{\mathrm{Sb}}}^{0}$	0	14.706	0	0	-5.626	-2.766	0	6.314
${V_{\mathrm{Sb}}}^{-1}$	-1	22.479	-4	0	-5.626	-2.766	0.397	10.484
${V_{\mathrm{Sb}}}^{-2}$	-2	28.255	-8	0	-5.626	-2.766	0.838	12.701
${V_{\mathrm{Sb}}}^{-3}$	-3	34.672	-12.001	0	-5.626	-2.766	1.552	15.831
${V_{\mathrm{Sb}}}^{-4}$	-4	39.227	-16.001	0	-5.626	-2.766	2.506	17.34
${V_{\mathrm{Sb}}}^{-5}$	-5	45.725	-20.001	0	-5.626	-2.766	3.833	21.165

Table S9: Calculated formation energies (ΔE^{form}) of extrinsic defects in F-doped Sb_2O_5 under Sb-rich condition, generated by DOPED.[5]

Defect	q	ΔE^{raw} (eV)	$q\epsilon_{VBM}$ (eV)	qE_F (eV)	$\Sigma\mu_{ref}$ (eV)	$\Sigma\mu_{formal}$ (eV)	E_{corr} (eV)	ΔE^{form} (eV)
${F_{O(c,\, C_1)}}^{1}$	1	-4.182	4	0	-3.937	1.55	0.133	-2.436
${F_{O(c,\, C_1)}}^{0}$	0	3.452	0	0	-3.937	1.55	0	1.065
${F_{O(c,\, C_1)}}^{-1}$	-1	11.115	-4	0	-3.937	1.55	-0.003	4.725
${F_{O(c,\, C_2)}}^{1}$	1	-4.015	4	0	-3.937	1.55	0.118	-2.284
${F_{O(c,\, C_2)}}^{0}$	0	3.623	0	0	-3.937	1.55	0	1.236
${F_{O(c,\, C_2)}}^{-1}$	-1	11.286	-4	0	-3.937	1.55	0.003	4.902
${F_{O(e,\, C_1)}}^{1}$	1	-3.378	4	0	-3.937	1.55	0.127	-1.638
${F_{O(e,\, C_1)}}^{0}$	0	4.243	0	0	-3.937	1.55	0	1.855
${F_{O(e,\, C_1)}}^{-1}$	-1	11.925	-4	0	-3.937	1.55	0.013	5.55
${F_i}^{+1}$	1	-7.884	4	0	3.586	2.308	0.183	2.194
${F_i}^{0}$	0	-1.294	0	0	3.586	2.308	0	4.6
${F_i}^{-1}$	-1	4.861	-4	0	3.586	2.308	0.023	6.778

References

(1) Jansen, M. Crystal Structure of Sb_2O_5. *Angewandte Chemie International Edition in English* **1978**, *17*, 137–137.

(2) George, J.; Petretto, G.; Naik, A.; Esters, M.; Jackson, A. J.; Nelson, R.; Dronskowski, R.; Rignanese, G.; Hautier, G. Automated Bonding Analysis with Crystal Orbital Hamilton Populations. *ChemPlusChem* **2022**, *87*, e202200123.

(3) Waroquiers, D.; George, J.; Horton, M.; Schenk, S.; Persson, K. A.; Rignanese, G.-

M.; Gonze, X.; Hautier, G. ıt ChemEnv: a fast and robust coordination environment identification tool. *Acta Crystallographica Section B* **2020**, *76*, 683–695.

(4) Ong, S. P.; Richards, W. D.; Jain, A.; Hautier, G.; Kocher, M.; Cholia, S.; Gunter, D.; Chevrier, V. L.; Persson, K. A.; Ceder, G. Python Materials Genomics (pymatgen): A robust, open-source python library for materials analysis. *Computational Materials Science* **2013**, *68*, 314–319.

(5) Kavanagh, S. R. Doped. `https://doi.org/10.21105/joss.06433`.
